# ATP signaling in the integrative neural center of *Aplysia californica*

**DOI:** 10.1038/s41598-021-84981-5

**Published:** 2021-03-09

**Authors:** János Györi, Andrea B. Kohn, Daria Y. Romanova, Leonid L. Moroz

**Affiliations:** 1 Department of Experimental Zoology, Centre for Ecological Research, Balaton Limnological Institute, 8237 Tihany, Hungary; 2 Whitney Laboratory for Marine Bioscience, University of Florida, St. Augustine, FL 32080 USA; 3 Institute of Higher Nervous Activity and Neurophysiology of RAS, Moscow, 117485 Russia; 4 Departments of Neuroscience and McKnight Brain Institute, University of Florida, Gainesville, FL 32610 USA

**Keywords:** Cellular neuroscience, Ion channels in the nervous system, Genetics of the nervous system, Molecular neuroscience, Neuroscience, Synaptic transmission, Neurotransmitters, Evolution

## Abstract

ATP and its ionotropic P2X receptors are components of the most ancient signaling system. However, little is known about the distribution and function of purinergic transmission in invertebrates. Here, we cloned, expressed, and pharmacologically characterized the P2X receptors in the sea slug *Aplysia californica*—a prominent neuroscience model. *Ac*P2X receptors were successfully expressed in *Xenopus* oocytes and displayed activation by ATP with two-phased kinetics and Na^+^-dependence. Pharmacologically, they were different from other P2X receptors. The ATP analog, Bz-ATP, was a less effective agonist than ATP, and PPADS was a more potent inhibitor of the *Ac*P2X receptors than the suramin. *Ac*P2X were uniquely expressed within the cerebral F-cluster, the multifunctional integrative neurosecretory center. *Ac*P2X receptors were also detected in the chemosensory structures and the early cleavage stages. Therefore, in molluscs, rapid ATP-dependent signaling can be implicated both in development and diverse homeostatic functions. Furthermore, this study illuminates novel cellular and systemic features of P2X-type ligand-gated ion channels for deciphering the evolution of neurotransmitters.

## Introduction

In addition to being the critical energy storage for every cell, ATP acts as one of the most ancient intracellular and intercellular signal molecules^1–3^. The possible involvement of ATP in signaling mechanisms was initiated in the 1920s by Drury and Szent-Gyorgyi^4^; and then in the 1950s by Holtons^5–7^, leading to the concept of purinergic transmission in the 1970s by Burnstock^3,8^. Eventually, in 1983, rapid ATP-gated ion currents were discovered in neurons^9,10^ and muscles^11^, and specific subtypes of the ligand-gated P2X receptors were identified in the 1990s^12–15^. Finally, the 3D structure of P2X receptors was revealed in 2009–2012^16,17^. These are distinctive trimeric ligand-gated channels showing a common architecture with acid-sensing ion channels but unrelated in their respective amino acid sequences^18^.

Comparative studies established that P2X-type receptors are broadly distributed across many eukaryotic lineages^1,19–21^, including most Metazoa^2,3^. Across all domains of life, ATP can operate as the *bona fide* ancient signal molecule (and a volume transmitter) associated with adaptive reactions to injury and damage^2,3,22^.

In selected evolutionary lineages, P2X receptors were secondarily lost. The list includes higher plants^3^, some bilaterians such as selected acoels^22^, arthropods, and nematodes. For example, *Drosophila* and *C. elegans* genomes have no P2X receptors, but other ecdysozoans such as *Daphnia*, the shrimp *Litopenaeus*, the tick *Boophilus*^23^, and tardigrades^24^ contain one receptor. Lophotrochosoans or Spiralia, including flatworms, also have one type of P2X receptor with shared pharmacological properties to mammals^25^. However, practically nothing is known about the functional roles of P2X receptors in the CNS and peripheral tissues of invertebrates and molluscs, in particular^3^. Mollusca is one of the most diverse animal phyla in terms of its morphological and biochemical adaptations.

The release of ATP from the central ganglia of the pond snail, *Lymnaea stagnalis,* was demonstrated^26^. Subsequently, P2X receptors were identified in this species with widespread expression across the CNS^27^ but unknown function(s).

Here, we show that ATP and its ligand-gated P2X receptors are components within chemosensory structures and the unique integrative neurosecretory center present in the CNS of the sea slug *Aplysia*—an important model for neuroscience^28,29^. Expression and pharmacology of P2X receptors in *Aplysia* confirms the preservation of evolutionary conserved bioenergetic reporter-sensor systems across animals and provides new tools to decipher homeostatic mechanisms in neuroendocrine systems and development.

## Results

### Identity, phylogeny, tissue-specific expression, and quantification of *Aplysia* P2X (*Ac*P2X) receptors

We identified and cloned a single *Aplysia* P2X receptor with two splice forms (GenBank#: NP_001191558.1, NP_001191559.1), which shared 92% identity. The predicted structure of the *Aplysia* P2X receptor reveals all major evolutionary conservative sites and posttranslational modifications (Supporting Information, Fig. S1), which are similar to its homolog in another gastropod, *Lymnaea*^27^. The genomic organization of the P2X receptors confirmed the overall evolutionary conservation of exons and intron–exon boundaries*.* The *Aplysia* P2X receptor is similar to a relatively compact vertebrate P2X4 receptor with similar numbers and length in exons, but this is not always found in other invertebrates (e.g., *Daphnia pulex* and *Nematostella vectensis* have 8 exons each). However, the overall similarity of the P2X4 receptor and the molluscan receptor intron/exon boundaries suggests that this receptor structure was present before divergence chordates and molluscs.

**Figure 1:**
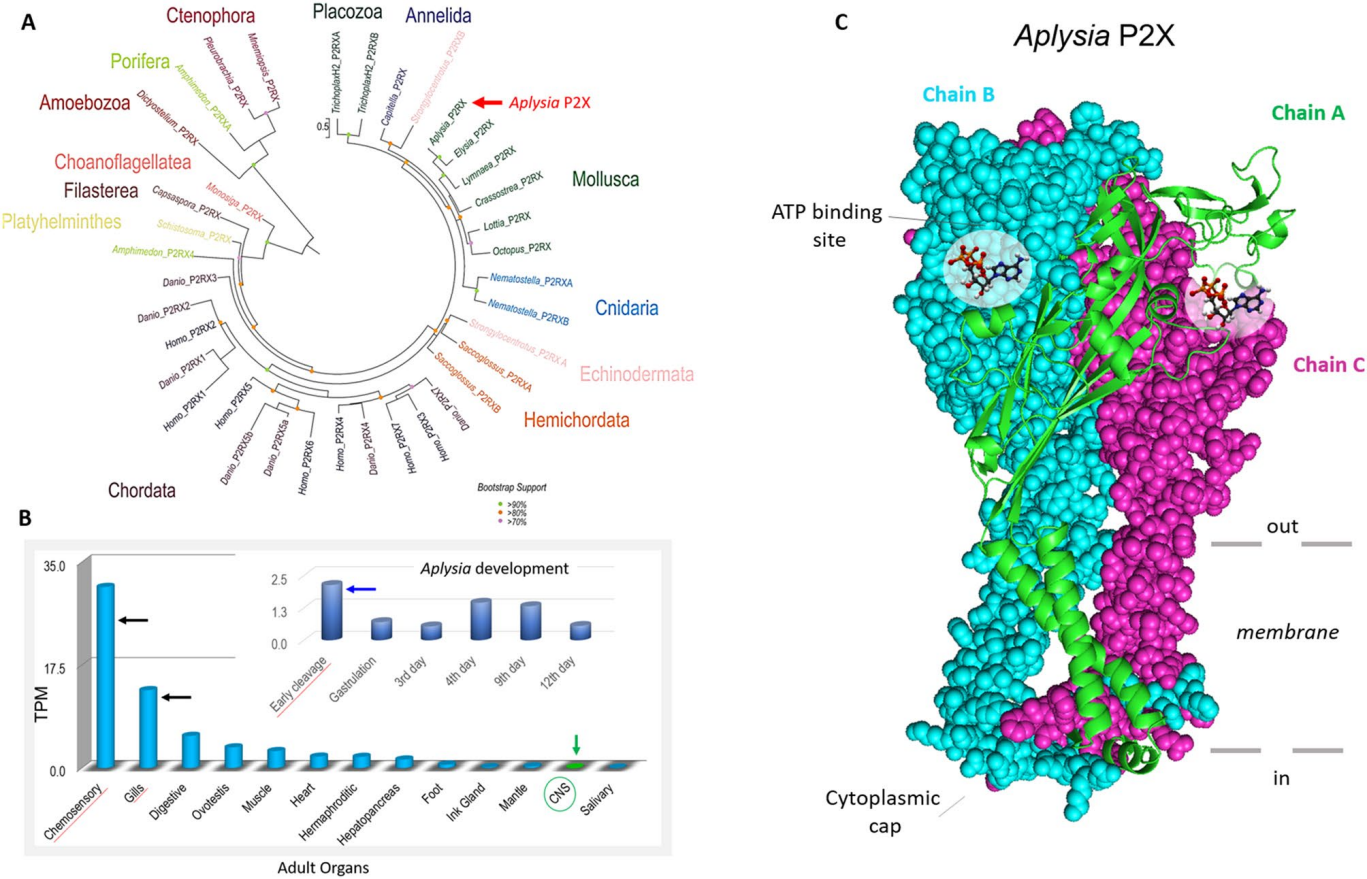
(**A**) Phylogenetic relationships of P2X and P2X-like receptors (P2RX). (**A**) A maximum likelihood (ML) phylogenetic tree of P2X receptors with the best-fit model (LG + G). Bootstrap support less than 70 omitted. Phylogenetically, the P2X predicted proteins cluster by phyla. P2X-type receptors are not unique to metazoans because they are detected in unicellular green algae *Ostreococcus tauri*^20^, the amoeba *Dictyostelium discoideum*^19^, the unicellular eukaryote *Monosiga brevicollis*^20^, as well as *Capsaspora owczarzaki*, and all these species, appear to have one P2X gene. Most of the non-bilaterians seem to have at least two P2X receptors (except for ctenophores, where only one receptor was detected). Lophotrochozoans, including the mollusc *Aplysia* and kins, appear to have one P2X receptor with different isoforms. The sea urchin and the acorn worm, *Saccoglossus,* both have at least two genes but numerous isoforms^1^. Humans^13^, as well as other chordates, appear to have seven unique P2X receptor genes^1^. See Supplementary Information, Table S1 for accession numbers of sequences used in this phylogenetic analysis. (**B**) Quantification of the expression of *P2X* receptors in the CNS, peripheral tissues, and developmental stages (insert) of *Aplysia*. The RNA-seq data represented as TPM (transcript per million) values^74–76^. The highest expression levels were detected in chemosensory areas (mouth areas and rhinophores), gills, and early developmental stages (Supplementary Information, Table S2 for RNA-seq projects). ***C***. 3D modeling for P2X receptor of *A. californica* (model PDB: 5svk). See text for details.

Figure 1A shows the phylogenetic relationships among P2X receptors with distinct events of gene duplications in the lineages leading to humans, zebrafishes, hemichordates, echinoderms, and basally-branched metazoans such as sponges, placozoans, and cnidarians (see Supporting Information, Table S1 for sequences used in this analysis). In contrast, representatives of molluscs (including *Aplysia*), annelids, and parasitic flatworms (*Schistosoma*) appear to have a single copy of a *P2X*-encoded gene that often form distinct phyletic clusters within a respective phylum. This reconstruction suggests a single *P2X* gene in the common metazoan ancestor with independent multiplication events in selected animal lineages. It primarily occurred within vertebrates, as reflected in the whole-genome duplication events early in the evolution of this group. Interestingly, some bilaterians such as the acoel *Hofstenia miamia*, insects, and nematodes^12^ secondarily lost the P2X receptors. This mosaic-type phyletic distribution likely illustrates different system constraints for recruiting P2X receptors to novel functions or preserving ancestral molecular mechanisms of purinergic signaling.

Next, we characterized the expression of the *P2X* receptors in *Aplysia* using a broad spectrum of RNA-seq data obtained from adult and developmental stages^30^ (see Supporting Information, Table S2 for project SRA accession numbers). The highest level of *AcP2X* expression was found in the chemosensory structures (the mouth area and rhinophores^31^) and the gill (Fig. 1B), which is also known as the chemosensory and respiratory organ. Expression of *AcP2X* receptors was detected in the majority of the peripheral organs in *Aplysia* as well as during the first developmental stages (Fig. 1B), where no neurons or specialized sensory cells exist. Thus, ATP could act as a paracrine messenger in early embryogenesis.

**Figure 2:**
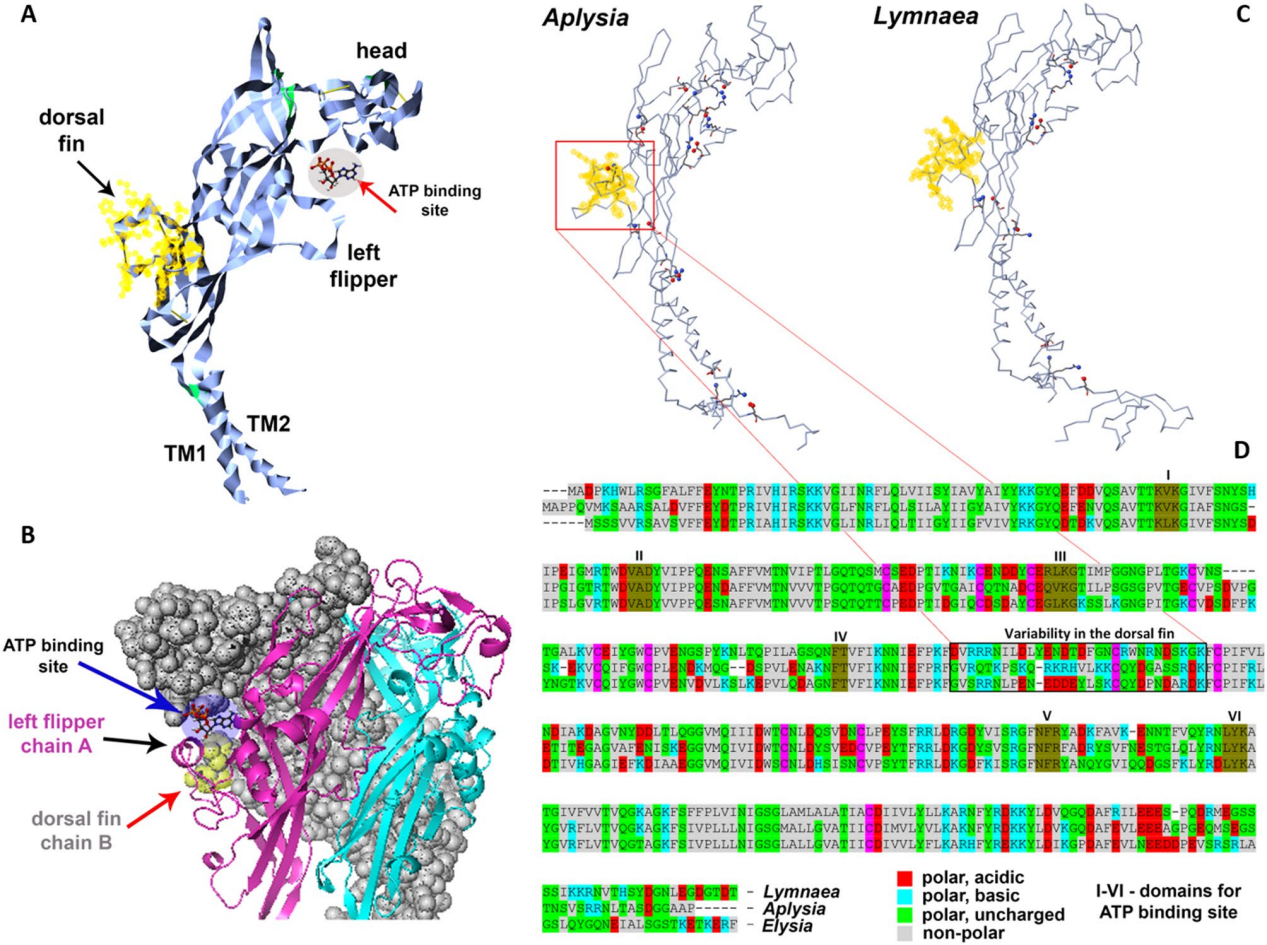
The organization of the P2X receptor in *Aplysia californica*. (**A**,**B**) Structural features of the P2X monomer models with regions recognized for mammalian homologs in crystallography studies^16,17^. TM1 and TM2 are transmembrane regions (see Fig. 1C). (**B**) 3D modeling of the trimeric P2X organization with suggested functional regions^16–18,77^. ‘Left flipper’ (chain A) and ‘dorsal fin’ (chain B) together with a head (chain A) of these chains form the ATP binding site (see also Fig. 1C). (**C**) Comparisons between *Aplysia* and *Lymnaea* receptors based on the predicted difference of salt bridges (yellow—‘dorsal fin’). (**D**) The alignment for P2X receptors in gastropod molluscs (*Aplysia californica*, *Lymnaea stagnalis* and *Elysia chlorotica*) with domain for the ATP binding site (brown—I-VI domains). Of note, *Aplysia* (middle in the alignment) has significantly less polar acidic and more polar basic amino acids in the ‘dorsal fin’ region [11pb:2pa] compared to other species (*Lymnaea* [7pb:6pa] and *Elysia* [5pb:8pa]), suggesting different kinetic and pharmacological properties of P2X receptors. In summary, there are 13 polar charged amino acids in the ‘dorsal fin’. See also Supplementary Figs. S1 and S2.

The freshwater pond snail *Lymnaea stagnalis* is the only know molluscan species with the biophysical characterization of its P2X receptors^27^. The *Aplysia* P2X receptor's structural organization was comparable to *Lymnaea*’s P2X (Figs. 1C, 2; and Fig. S2 Supplementary Information) but with noticeable differences in their predicted ATP binding and other regulatory sites suggesting potentially different biophysical properties. These differences are also evident in the 3D models for related molluscan species (Fig. 2 and Fig. S2, Supplementary Information). Specifically, the differences between *Aplysia* and *Lymnaea* were found in the region of the P2X receptors known as the ‘dorsal fin’^16,32^. The crystallography analysis^16^ indicated that ATP binding causes rearrangement of the ‘head’, ‘right flipper’, and ‘dorsal fin’ domains (Fig. 2A,B). These conformational changes occur both around the ligand (ATP) binding site and between subunits^16,33^. Thus, the observed variability of amino acid sequences in these regions between *Aplysia* and *Lymnaea* suggests that marine and freshwater organisms are distinct in the kinetic and pharmacological properties of their respective P2X receptors. This possibility was experimentally tested, as we reported below.

### Expression of *Ac*P2X in *Xenopus* oocyte confirms the evolutionary conservation of kinetic and pharmacological parameters

**Figure 3:**
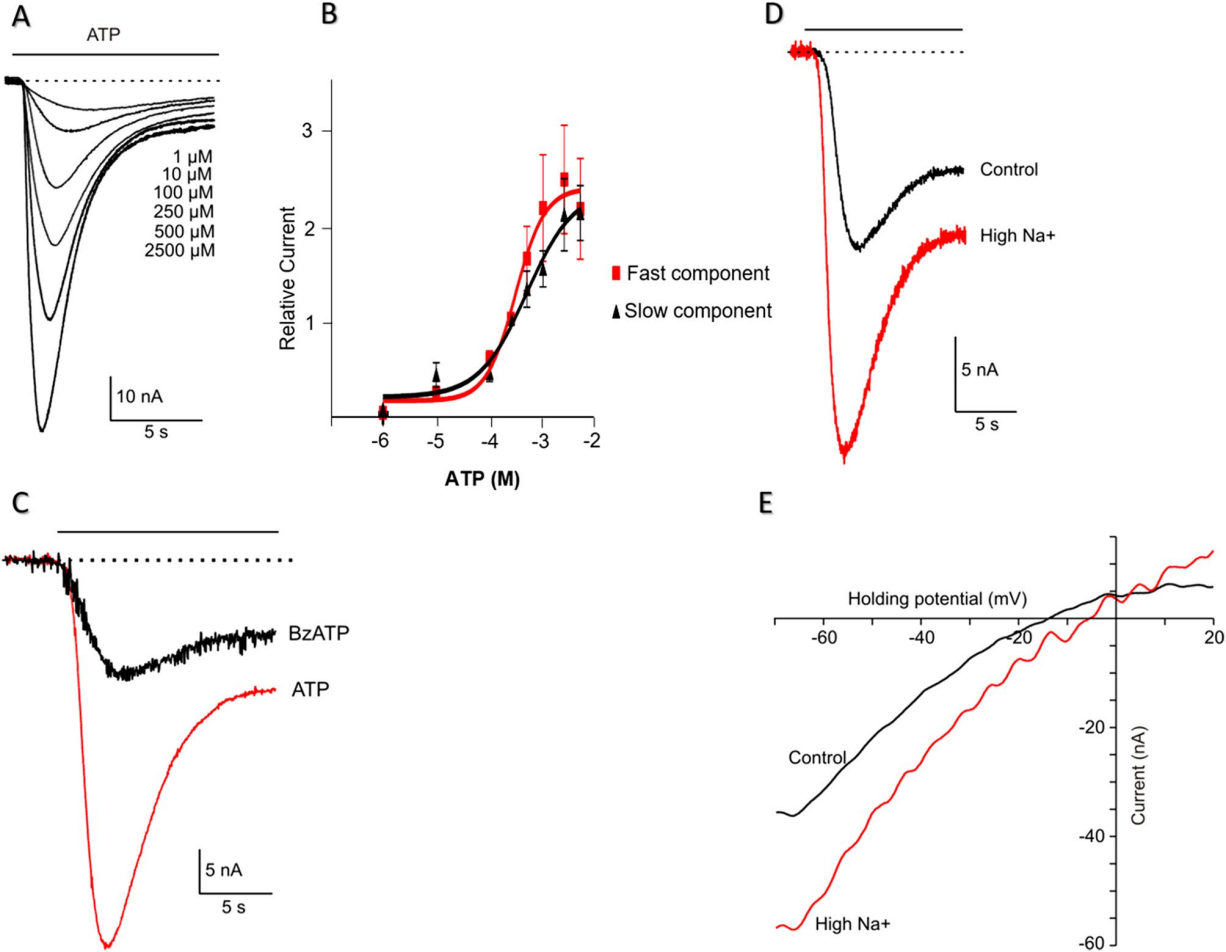
Functional expression of recombinant *Ac*P2X receptors in *Xenopus* oocytes. (**A**) Examples of currents recorded in response to different concentrations of ATP (HP = − 70 mV, agonist application indicated by the solid line*)*. (**B**) Dose–response curves for ATP receptor activation. Mean currents were normalized to the response given by 250 μM ATP (*n* = 7, oocytes). Serially increasing concentrations of ATP were applied to oocytes for 15 s at 6-min intervals. Symbols represent mean ± S.E. Continuous line for *ATP* represents data fitted using the equation I = I_max_/[1 + (IC50/L)*nH*], where I is the actual current for a ligand concentration (L), *nH* is the Hill coefficient, and I_max_ is the maximal current (IC50_fast_ = 306.0 μM, IC50_slow_ = 497.4 μM; *nH*_*fast*_ = *1.58, nH*_*slow*_ = *0.97*). ***C***. Two-electrode voltage-clamp recordings from oocytes expressing *Ac*P2X receptors. Representative inward currents recorded in response to ATP (red trace) and the 250 μM of Bz-ATP (HP = − 70 mV, application indicated by the solid line) (*n* = 8, oocytes). (**D**) Recordings of ATP-induced current (250 μM, ATP) in the presence of normal [Na^+^] (96 mM) and with elevated extracellular Na^+^ (144 mM; red trace); HP = -70 mV(*n* = 6, oocytes). (**E**) Ramp voltage-clamp protocol from − 70 mV HP to 20 mV in the presence of 250 μM ATP. The plots of the subtracted current (a current in the presence of ATP minus the current in the absence of ATP) against voltage during the ramp. The red trace—high [Na^+^], 144 mM. According to the Nernst equation, the reversal potential was shifted by 10.2 ± 1.3 mV to the + direction of the *holding* potential (n = 6, oocytes).

ATP elicited an inward current in a concentration-dependent manner in oocytes injected with *AcP2X* (Fig. 3A)_._ EC50s (concentrations of a drug that gives half-maximal response) were determined for both the fast- (0-1seconds) and the slow component of current with continuous application of ATP. The EC50 for the fast component was 306.0 µM with a 1.58 Hill coefficient, and for the slow component, 497.4 µM with a 0.97 Hill coefficient (n = 7, oocytes, Fig. 3B). The second application of the agonist, with a recovery time of 6 min, generated a 15–30% reduction in peak amplitude and is indicative of the rundown observed in other P2X receptor subunits. The response to 250 µM ATP produced a mean peak amplitude of 31.3 nA ± 3.8 nA and a time to a peak value of 2.76 ± 0.21 s (n = 19) with a holding membrane potential of − 70 mV (Fig. 3C). The ATP analog, 2′,3′-O-(4-Benzoylbenzoyl) adenosine 5′-triphosphate (Bz-ATP^34^) gave a partial response at 20% of the ATP response (n = 8, oocytes, Fig. 3C). There were no UTP and ADP responses within the same range of concentrations (data not shown).

The current–voltage relationship was investigated in the presence of elevated (144 mM) and low extracellular NaCl (96 mM) concentrations (n = 6, oocytes, Fig. 3D). A reversal potential was determined by applying a ramp protocol from − 70 to 20 mV in high and normal Na^+^ with 250 µM of ATP (Fig. 3E). According to the Nernst equation, the reversal potential was 13.9 mV and shifted by + 10.2 ± 1.3 mV to positive holding in high sodium solution (n = 6, oocytes).

**Figure 4:**
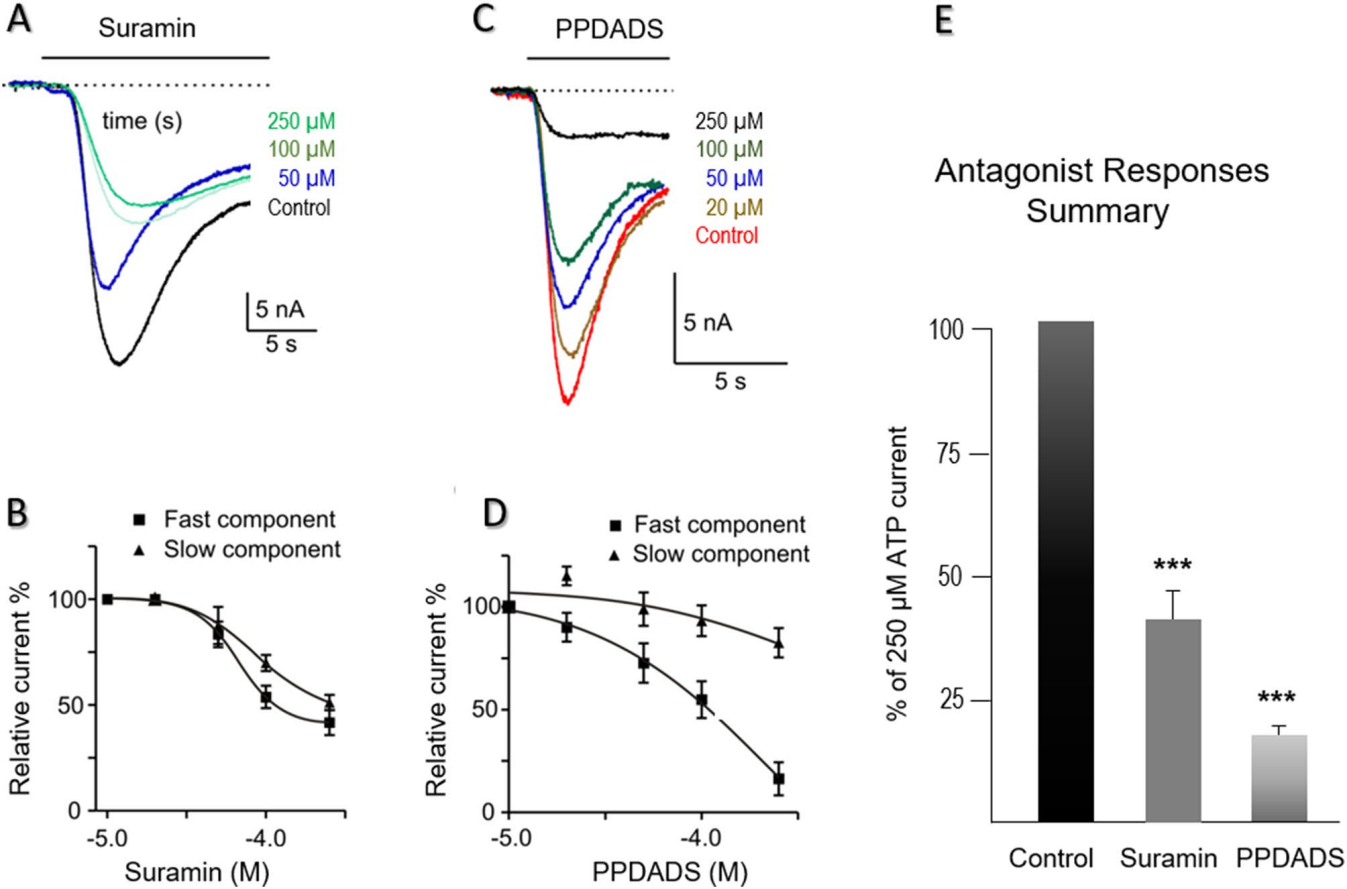
Pharmacology of *Ac*P2X receptors in *Xenopus* oocytes. (**A**) Example of currents induced by 250 μM ATP. ATP was applied to oocytes for 15 s, in the presence of varying concentrations of suramin (HP = − 70 mV). (**B**) Mean responses to 250 μM ATP in the presence of 1–250 μM suramin. There was a suramin-resistant component of the *Ac*P2X current. Symbols represent mean ± S.E *n* = 7, oocytes). (**C**) Traces recorded in response to 250 μM ATP in the presence of varying concentrations of the second antagonist, PPADS (concentrations are shown in μM, and all applications are indicated by the solid lines) (n = 7, oocytes). (**D**) Mean responses to 250 μM of ATP in the presence of the PPADS (a fast component of responses—closed squares, slow component—triangles). PPADS was an effective antagonist in the range of 1–250 μM. Fitting of the data using the sigmoidal dose–response curve by a continuous line, IC50_fast_ = 211.2. Symbols represent the mean ± S.E (n = 7, oocytes). (**E**) The suramin proved to be a more effective blocker of the ATP-activated channels among the two antagonists tested. A chart of mean currents (% of 250 µM ATP response) in the presence of 250 µM Suramin and 250 µM PPADS. Mean currents were normalized to the response given by 250 μM of ATP. Symbols represent mean ± S.E; statistically significant differences (Student’s *t*-test) from control (P < 0.05) are indicated by asterisks (***) above the bars (n = 7, oocytes).

P2X antagonist suramin^34^ inhibited ATP responses in a concentration-dependent manner (Fig. 4A,B; n = 7, oocytes). Another P2X antagonist, pyridoxal-phosphate-6-azophenyl-2′,4-disulfonic acid (PPADS^34^), also inhibited the response of ATP on *Ac*P2X in a concentration-dependent manner (Fig. 4C,D). However, the application of PPADS produced a greater block than the suramin (Fig. 4E). Mean current responses to 250 µM ATP in the range of 1–250 µM PPADS generated an IC50 = 211.2 µM for the fast component, but the slow component could not be calculated (n = 7, oocytes, Fig. 4D). The second splice form of *AcP2X*_*b*_was also expressed in oocytes producing currents very similar to the first isoform *AcP2X* described above; however, it resulted in much smaller (and unstable) responses (data not shown).

### P2X receptors are expressed in unique populations of neurosecretory cells of *Aplysia*

Interestingly, the CNS has the overall lowest *P2X* gene expression (Fig. 1B). This situation might be analogous to the recruitment of purinergic signaling in the chemosensation within the mammalian brain^35^, suggesting a distinct and relatively small population of ATP-sensing cells. We tested this hypothesis.

**Figure 5:**
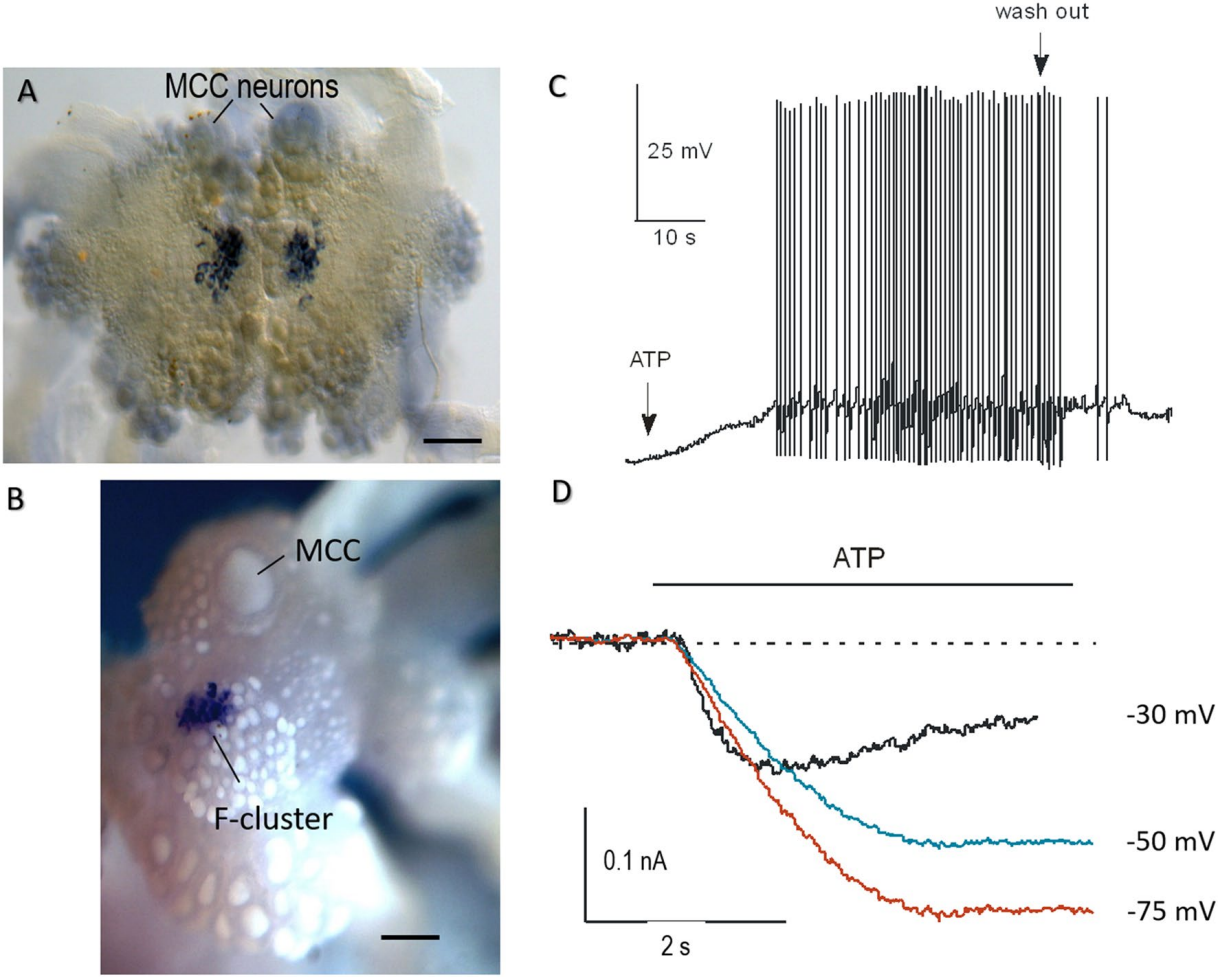
Distribution of *AcP2X* in the CNS of *Aplysia* and the effect of ATP on *Aplysia* F-cluster neurons. (**A**,**B**) *AcP2X* is expressed in neurons of the cerebral F-cluster (in situ hybridization). A pair of giant serotonergic feeding interneurons (MCC) are indicated by arrows. (**A**) The preparation embedded in a mounting media. (**B**) The cerebral ganglion was photographed in 100% ethanol. Scale 300 µm. (**C**) Current-clamp recording from F-cluster neurons in the intact CNS. Bath application of ATP (2.0 mM) caused an excitatory response with spiking activity (2–5 mV depolarization with a burst of the action potentials), and full recovery following washout (indicated by arrows) (n = 7, neurons). (**D**) Voltage-clamp recording from F-cluster neuron. Raw traces were recorded in response to 2.0 mM of ATP at three holding potentials (agonist application indicated by the line).

*AcP2X* was explicitly expressed in two symmetrical subpopulations localized in the F-cluster of the cerebral ganglion of *Aplysia*^36,37^. Each subpopulation contained about 25–40 cells (30–60 μm diameter, Fig. 5A,B)^36^. As illustrated in Fig. 5A, but not as in the present version Fig. 1A the labeled cells are not homogenous and might represent different subpopulations (larger and middle-size cells neurons^36^, and some of these neurons can be insulin-containing^37^). Application of 2 mM ATP to these neurons elicited a 2–5 mV depolarization (3.1 mV ± 2.2 mV n = 11), action potentials, and these effects were reversible (Fig. 5C) and voltage-dependent (Fig. 5D), consistent with the pharmacological properties of *Aplysia*’s P2X receptors expressed in oocytes. Neurons from neighboring cerebral clusters that were negative for *AcP2X* by in situ hybridization showed no response to as high as 10 mM ATP concentration (n = 31). These tests confirmed that P2X receptors in F-cluster neurosecretory cells are functional.

## Discussion

As the central bioenergetic currency, the intracellular concentrations of ATP reach 1–10 mM with multiple mechanisms of its extracellular release across all domains of life^2^. In the molluscan CNS, the baseline level of ATP release can be increased following depolarization and serotonin application, suggesting that ATP can act as an endogenous fast neurotransmitter^26^. The presence of ionotropic P2X ATP-gated cationic channels in peripheral chemosensory structures (together with profound serotonergic innervations of these structures in molluscs^38–44^) and the CNS (Fig. 1B) further support this hypothesis. However, we also reported *AcP2X* receptor expressions early in development, suggesting that ATP might be a paracrine signal molecule controlling cleavage and differentiation.

The purinergic sensory transmission is widespread in mammals^35^ and might have deep evolutionary roots^2,3,45^. Mammalian P2X receptors^13,46^ are comparable to their homologs in *Aplysia* based on their sequence and kinetic parameters but have a lower sensitivity to ATP. As a part of future directions in comparative biophysical studies, it would be important to perform targeted mutagenesis of the molluscan P2X receptors to precisely identify amino acids responsible for the observed differences among mammalian and invertebrate P2X receptors.

The overall kinetic and pharmacological parameters of *Aplysia* P2X receptors are also similar to those described both in the closely related *Lymnaea*^27^ and distantly related *Schistosoma*^25^. However, the *Lymnaea* P2X receptor showed much higher sensitivity (EC50 is in µmolar range) to ATP than *Aplysia*, consistent with predicted structural differences of P2X receptors across species (Fig. 2). Suramin and PPADS both inhibited the ATP evoked responses in other species^23–25,27^, but in *Aplysia,* it occurred in a narrower range (10–250 µM) than in *Lymnaea* (0.1–250 µM). Suramin and PPADS are known as pan-P2X blockers. Thus, it might be expected that novel and, perhaps, more selective antagonists could exist for the *Aplysia* receptor subtype.

Ecdysozoan P2X receptors are also relatively diverse (although detailed mutagenesis studies have not been performed in any representatives of this largest metazoan lineage). In contrast to *Aplysia*, the tick P2X receptor displayed a very slow current kinetics and little desensitization during ATP application^23^. The tardigrade P2X receptor^24^ had a relatively low sensitivity for ATP (EC50 ~ 44.5 μM), but fast activation and desensitization kinetics—similar to *Aplysia*.

*Aplysia* P2X receptors exhibit a distinct phenotype, having a moderate ATP sensitivity (compared to the freshwater *Lymnaea* and mammals) but faster kinetics than some ecdysozoans. These “hybrid features” might be related to the marine ecology of *Aplysia* with a broader range of environmental changes. The agonist potencies (ATP and BzATP) in the cloned *Aplysia* P2X do not match any known P2X receptor subtype. It suggests that there was a specific diversification of P2X receptors in the lineage leading to *Aplysia*.

The currents recorded from the *Aplysia* F-cluster neurons do not fully resemble the currents recorded from the oocytes. They do not desensitize and get bigger as the membrane potential hyperpolarizes. These observations also suggest that either expression in *Xenopus* (as the freshwater animal with different osmolarity) modify the properties of native channels or implicate additional regulatory mechanisms for the *Aplysia* P2X receptors in the intact CNS. Additional integrative analyses would be needed to biophysically determine the channel properties and physiological role of *Ac*P2X receptors.

Interestingly, the abundance of *P2X* receptors in the *Aplysia* chemosensory systems (such as mouth areas, rhinophores, and gills) correlates with the expression of nitric oxide synthase^47^, suggesting interactions of these afferent pathways in the control of feeding and respiration. *Aplysia* might also detect environmental ATP from bacterial and algal (food) sources^2^ as in some other studied marine species, including lobsters^48^.

The P2X receptor is one of many different receptors in the purinergic signaling complex. The ‘purinome’ includes P2 receptors, both ionotropic (P2X) and metabotropic (P2Y), P1 adenosine receptors, adenosine transporters ectonucleotidases^49,50^. Of note, the *Aplysia* genome has no *P2Y* receptors^51^. Still, it has twenty-four *P1* adenosine receptors that are also putative purinergic G protein-coupled receptors, compared to the human genome that contains eight *P2Y* receptors and four *P1* adenosine receptors^51^. Besides the purinergic receptors, there are two gene families related to purinergic signaling: adenosine transporter proteins, equilibrative (*ENT*) and concentrative nucleoside transporters (*CNT*)^52–54^. The *Aplysia* genome has no *CNT*s but contains five predicted *ENTs,* whereas the human genome has three *CNTs* and four *ENTs.* A putative vesicular nucleotide transporter*, VNUT* (SLC17a)^55,56^ was detected in the *Aplysia* genome *(*Solute carrier family 17 member 9, e-value = 0*)*^57^. However, it should be noted this predicted *Aplysia* gene (XP_005101701.3) is a tentative annotation without experimental validation of its specific nucleotide affinities (e.g., ATP). Its ortholog, *VNUT*, has only been functionally characterized in humans and mice^55^. XP_005101701.3 would be an essential gene for future experimental analysis of expression and selectivity determination in *Aplysia*.

The *Aplysia* genome has a representative complement of ectoenzymes (cell surface enzymes that can hydrolyze nucleosides and nucleotides extracellularly^58,59^), including the enzymes of the nucleoside triphosphate diphosphohydrolase (ENTPDase) family, the nucleotide pyrophosphatase/phosphodiesterase (ENPP) family, ecto-5′-nucleotidase (5NTD), alkaline phosphatase (AP), adenosine deaminase (ADA) and purine nucleoside phosphorylase (PNP)^59^. The presence of this type of extracellular enzymes can restrict the volume type transmission mediated by ATP, and purinergic signaling in the CNS might be more localized in 3D space and time.

The purinergic signaling system is prominent and widespread in the human brain. Yet, in the CNS of *Aplysia*, the detectable expression of the ionotropic P2X receptor is apparently confined to the F-cluster, emphasizing the integrative role of this neurosecretory center. F-cluster also has insulin-containing neurons^36,37^, likely associated with the systemic control of growth and, subsequently, reproduction. The release of the *Aplysia* insulin can decrease the level of glucose in the hemolymph^37^. Moreover, some F-cluster neurosecretory cells are electrically coupled^36^, which help synchronize their discharges and, eventually, insulin secretion. It is known that ATP can also be released from gap junctions (innexins) and during synaptic exocytosis^2^. Thus, we can propose that P2X-induced neuronal depolarization of insulin-containing neurons provides positive purinergic feedback, sustaining this multifunctional integrative center's excitability and secretory activity in *Aplysia*-related gastropods.

The potential functional roles of many differentially expressed genes in the F-cluster can be determined in the future. Their involvements in control of multiple behaviors, immunity is evidenced by a broad array of respective genes uniquely or differentially expressed in F-cluster (our recent RNA-sed data revealed unexpected diversity of specific transcripts controlling pain, injury, toxins, immunity [interleukins], feeding, and energetics [insulins], reproduction and potentially some novel functions [ecdysone-type receptors], etc.—LLM, ABK, unpublished data). This is the reason to view this cluster as one of the top-level integrative centers in the animal with the broadest spectrum of secretory peptides and multiple receptors, including P2X receptors.

This study also clearly illustrates that genomic/molecular predictions have to be experimentally validated as we performed here with the *Aplysia* P2X receptors. Indeed, many observed differences in kinetics and pharmacological properties of these *Aplysia* receptors (compared to other species) cannot be deduced from the sequence information. It further stresses physiological experiments' value in analyzing predictions of emerging massive comparative genomics/transcriptomics datasets.

## Materials and methods

### Animals and molecular analyses of *Aplysia* AcP2X

*Aplysia californica* (60–100 g) were obtained from the National Resource for *Aplysia* at the University of Miami. Animals were anesthetized by injection of 60% (volume/body weight) isotonic MgCl_2_ (337 mM) prior to the removal of the CNS. The ganglia were then pinned to a sylgard dish in artificial seawater (ASW: 460 mM NaCl, 10 mM KCl, 55 mM MgCl_2_, 11 mM CaCl_2_, 10 mM HEPES, pH = 7.6), and the cells were exposed by the mechanical removal of the overlying sheath with fine forceps.

### Cloning

The original sequences were generated using RNA-seq profiling^60–63^. Details for RNA extraction and cDNA library construction have been described^60–62,64^.

Both 5′ and 3′ RACE were performed to obtain the full-length coding sequence. Full-length CDS sequence for *Ac*P2X (GenBank# NP_001191558.1) was obtained using terminal primers: 5′-ATGGCTCCACCACAAGTCATGAAG-3′ and 5′-AAGCATCAAGGTGCGGCTCCTCCATCAC-3′. Amplified PCR product was cloned into pCR4-TOPO (Cat#K4575-01, LifeTechnologies). Four clones were isolated and sequenced together with a splice variant, *Ac*P2X_b_ (GenBank# NP_001191559.1), and the full-length CDS for this isoform was also cloned and sequenced. Since there appears to be only one P2X gene in the *Aplysia* genome (GCF_000002075.1), we will designate the predicted protein as *Ac*P2X.

### In situ hybridization

The original sequences were obtained from *Aplysia* RNA-seq profiling^60–63^. We used the same protocols for whole-mount in situ hybridization as reported elsewhere^65,66^ with a specific probe for the validated *AcP2X*. The two isoforms of *AcP2X* vary by a 147 base deletion/insertion and would be difficult to distinguishable by in situ. The antisense probe was generated by digestion of the *AcP2X* plasmid with Not I (Cat#R0189s, New England Biolabs Inc.) then transcribed with T_3_ polymerase from the DIG RNA Labeling Kit (Cat#11175025910, Roche Diagnostics). The control sense probe was produced by the same protocol but used Pme1 (Cat# R0560s, New England Biolabs Inc.) for digestion and T_7_ polymerase for transcription.

Expression of *AcP2X* was investigated in central ganglia of 8 experimental and 2 control CNS preparations; additional controls were reported elsewhere^60,66^. Control in situ hybridization experiments with full length ‘sense’ probes revealed no specific or selective localization in the CNS under identical conditions and labeling protocols. Images were acquired with a Nikon Coolpix 4500 digital camera mounted on an upright Nikon Optiphot-2 microscope.

### RNA-seq and quantification

The isolation of specific cells, RNA extraction, and preparation of cDNA for RNA-seq analysis as well as transcriptome annotation was performed using the same methods as reported elsewhere^60–62,64^.

Expression levels of transcripts were calculated using the normalization method for RNA-seq—Transcripts Per Million (TPM)^67^. Mapping was performed in the STAR (2.3.0)/feature Counts analysis with the values obtained from the Bowtie2/Tophat pipeline^68^. The mapped reads were summarized and counted within the R statistical programming language. Supplementary Table S2 contains a list of RNA-seq projects and their corresponding SRA accession number.

### Sequence analysis and phylogeny

Sequences were obtained through BLAST search across both Metazoans and non-metazoan groups and aligned using the MUSCLE or ClustalX ver. 2.1 programs with default parameters. Protein domains and motifs were obtained from Prosite49 and SMART50 databases. A maximum likelihood (ML) tree with the best-fit model (LG + G) was constructed using MEGA X51 and 10,000 iterations.

### Protein modeling

The reconstruction of 3D-structures of the P2X receptor from *Aplysia californica* (NP_001191558.1, GenBank, NCBI) and *Lymnaea stagnalis* (AFV69113.1, Genbank, NCBI) was based on pdb ID: 5svk (open state) and 4dwo (closed state) modeling^69^. Alternative models of the same P2X receptors were generated using PyMol (The PyMol Molecular Graphics System, Version 1.8.6.0 Schrödinger, LLC) and Phyre2 software^69–72^.

## Electrophysiology

### Oocytes recordings

RNA preparation for oocyte injections and oocyte maintenance are described above. The oocyte recording bath was in ND96 medium (96 mM NaCl, 2 mM KCl, 1 mM MgCl_2_, 1.8 mM CaCl_2_, and 5 mM HEPES, pH = 7.4), or with the 1.8 mM CaCl_2_ being replaced by 1.8 mM BaCl_2_. Ba^2+^ was used in oocytes experiments only to block the endogenous chloride and potassium currents in *Xenopus* oocytes*.* Whole-oocyte currents were recorded by two-electrode voltage clamp (GeneClamp500B, Axon Instruments, Foster City, CA, USA) using microelectrodes made of borosilicate glass (WPI, USA) with a resistance of 0.5–1 MΩ when filled with 2.5 M KCl. Currents were filtered at 2 kHz and digitally sampled at 5 kHz with a Digidata 1320B Interface (Axon Instruments, CA). Recording and data analysis were performed using pCLAMP software version 8.2 (Axon Instruments). For data acquisition and clamp protocols, the amplifiers were connected via a Digidata 1320B AD/DA converter (Axon, USA) to an AMD PC with pClamp 8.2 voltage-clamp software (Axon, USA). Unfiltered signals were sampled at 10 kHz and stored digitally.

Data are presented as mean ± S.E. using Student’s paired *t*-test. Concentration–response data were fitted to the equation I = I_max_/[1 + (EC50/L)^*nH*^], where I is the actual current for a ligand concentration (L), *n*_*H*_ is the Hill coefficient, I_max_ is the maximal current, and EC50 is the concentration of agonist evoking 50% the maximum response. Respectively, the **IC50** is the concentration of an inhibitor where the response (or binding) is reduced by half.

To compute the reversal potential for sodium, the Nernst equation used; Vj = (RT)/(zF)ln(c1/c2) where R is the gas constant 1.98 cal K^−1^ mol^−1^, F is the Faraday constant 96,840 C/mol, T is the temperature in ^o^K and z is the valence of the ion.

### In situ recordings

Voltage- and current-clamp experiments were carried out on identified F-cluster neurons in intact nervous systems of *Aplysia*^36^. ~ 0.5 mL bath was perfused with solutions (artificial sea water) using a gravity-feed system and a peristaltic pump, and solution exchanges were performed by VC-6 six-channel valve controller (Warner Inst., USA). Conventional two-electrode (3–10 MΩ) voltage-clamp techniques (Axoclamp2B, TEVC mode) were employed to measure agonist-activated currents as reported^73^ at room temperature(20 ± 2 °C). To characterize membrane and action potentials, we used a bridge mode of Axoclamp2B with borosilicate microelectrodes (tip resistance: 10–18 MΩ, with 0.5 M KCl, 2 M K-Acetate, and 5 mM HEPES, pH = 7.2).

## Supplementary Information

Supplementary Information

## Data Availability

All data are contained within the article as well in Supplementary Information.

## References

[1] Hou Z, Cao J (2016). Comparative study of the P2X gene family in animals and plants. Purinergic Signal.

[2] Verkhratsky A, Burnstock G (2014). Biology of purinergic signalling: its ancient evolutionary roots, its omnipresence and its multiple functional significance. BioEssays.

[3] Verkhratsky, A. Early evolutionary history (from bacteria to hemichordata) of the omnipresent purinergic signalling: A tribute to Geoff Burnstock inquisitive mind. *Biochem Pharmacol*, 114261, doi:10.1016/j.bcp.2020.114261 (2020). 10.1016/j.bcp.2020.114261 33011161

[4] Drury AN, Szent-Gyorgyi A (1929). The physiological activity of adenine compounds with especial reference to their action upon the mammalian heart. J. Physiol..

[5] Holton P (1959). The liberation of adenosine triphosphate on antidromic stimulation of sensory nerves. J. Physiol..

[6] Holton FA, Holton P (1954). The capillary dilator substances in dry powders of spinal roots; a possible role of adenosine triphosphate in chemical transmission from nerve endings. J. Physiol..

[7] Holton FA, Holton P (1952). The vasodilator activity of spinal roots. J. Physiol..

[8] Burnstock G (1972). Purinergic nerves. Pharmacol. Rev..

[9] Krishtal OA, Marchenko SM, Pidoplichko VI (1983). Receptor for ATP in the membrane of mammalian sensory neurones. Neurosci. Lett..

[10] Jahr CE, Jessell TM (1983). ATP excites a subpopulation of rat dorsal horn neurones. Nature.

[11] Kolb HA, Wakelam MJ (1983). Transmitter-like action of ATP on patched membranes of cultured myoblasts and myotubes. Nature.

[12] Surprenant A, Buell G, North RA (1995). P2X receptors bring new structure to ligand-gated ion channels. Trends Neurosci..

[13] North RA (2002). Molecular physiology of P2X receptors. Physiol. Rev..

[14] Brake AJ, Wagenbach MJ, Julius D (1994). New structural motif for ligand-gated ion channels defined by an ionotropic ATP receptor. Nature.

[15] Valera S (1994). A new class of ligand-gated ion channel defined by P2x receptor for extracellular ATP. Nature.

[16] Kawate T, Michel JC, Birdsong WT, Gouaux E (2009). Crystal structure of the ATP-gated P2X(4) ion channel in the closed state. Nature.

[17] Hattori M, Gouaux E (2012). Molecular mechanism of ATP binding and ion channel activation in P2X receptors. Nature.

[18] Baconguis I, Hattori M, Gouaux E (2013). Unanticipated parallels in architecture and mechanism between ATP-gated P2X receptors and acid sensing ion channels. Curr. Opin. Struct. Biol..

[19] Fountain SJ (2007). An intracellular P2X receptor required for osmoregulation in *Dictyostelium discoideum*. Nature.

[20] Fountain SJ, Cao L, Young MT, North RA (2008). Permeation properties of a P2X receptor in the green algae *Ostreococcus tauri*. J. Biol. Chem..

[21] Cai X (2012). P2X receptor homologs in basal fungi. Purinergic Signal.

[22] Moroz LL, Romanova DY, Kohn AB (2021). Neural versus alternative integrative systems: molecular insights into origins of neurotransmitters. Philos. Trans. R Soc. Lond. B Biol. Sci..

[23] Bavan S (2011). The penultimate arginine of the carboxyl terminus determines slow desensitization in a P2X receptor from the cattle tick *Boophilus microplus*. Mol. Pharmacol..

[24] Bavan S, Straub VA, Blaxter ML, Ennion SJ (2009). A P2X receptor from the tardigrade species *Hypsibius dujardini* with fast kinetics and sensitivity to zinc and copper. BMC Evol. Biol..

[25] Agboh KC, Webb TE, Evans RJ, Ennion SJ (2004). Functional characterization of a P2X receptor from *Schistosoma mansoni*. J. Biol. Chem..

[26] Gruenhagen JA, Lovell P, Moroz LL, Yeung ES (2004). Monitoring real-time release of ATP from the molluscan central nervous system. J. Neurosci. Methods.

[27] Bavan S, Straub VA, Webb TE, Ennion SJ (2012). Cloning and characterization of a P2X receptor expressed in the central nervous system of *Lymnaea stagnalis*. PLoS ONE.

[28] Moroz LL (2011). Aplysia. Curr. Biol..

[29] Kandel ER (2001). The molecular biology of memory storage: a dialogue between genes and synapses. Science.

[30] Heyland A, Vue Z, Voolstra CR, Medina M, Moroz LL (2011). Developmental transcriptome of *Aplysia californica*. J. Exp. Zool. B Mol. Dev. Evol..

[31] Moroz LL (2006). Localization of putative nitrergic neurons in peripheral chemosensory areas and the central nervous system of *Aplysia californica*. J. Comp. Neurol..

[32] Gonzales EB, Kawate T, Gouaux E (2009). Pore architecture and ion sites in acid-sensing ion channels and P2X receptors. Nature.

[33] Mansoor SE (2016). X-ray structures define human P2X(3) receptor gating cycle and antagonist action. Nature.

[34] North RA, Surprenant A (2000). Pharmacology of cloned P2X receptors. Annu. Rev. Pharmacol. Toxicol..

[35] Gourine AV, Llaudet E, Dale N, Spyer KM (2005). ATP is a mediator of chemosensory transduction in the central nervous system. Nature.

[36] Rubakhin SS, Li L, Moroz TP, Sweedler JV (1999). Characterization of the *Aplysia californica* cerebral ganglion F cluster. J. Neurophysiol..

[37] Floyd PD (1999). Insulin prohormone processing, distribution, and relation to metabolism in *Aplysia californica*. J. Neurosci..

[38] Moroz LL, Sudlow LC, Jing J, Gillette R (1997). Serotonin-immunoreactivity in peripheral tissues of the opisthobranch molluscs *Pleurobranchaea californica* and *Tritonia diomedea*. J Comp Neurol.

[39] Sudlow LC, Jing J, Moroz LL, Gillette R (1998). Serotonin immunoreactivity in the central nervous system of the marine molluscs *Pleurobranchaea californica* and *Tritonia diomedea*. J. Comp. Neurol..

[40] Hawkins RD (1989). Localization of potential serotonergic facilitator neurons in *Aplysia* by glyoxylic acid histofluorescence combined with retrograde fluorescent labeling. J. Neurosci..

[41] Kistler HB (1985). Distribution of serotonin-immunoreactive cell bodies and processes in the abdominal ganglion of mature *Aplysia*. J. Neurosci..

[42] Longley RD, Longley AJ (1986). Serotonin immunoreactivity of neurons in the gastropod *Aplysia* californica. J. Neurobiol..

[43] Martinez-Rubio C, Serrano GE, Miller MW (2009). Localization of biogenic amines in the foregut of *Aplysia californica*: catecholaminergic and serotonergic innervation. J. Comp. Neurol..

[44] Wertz A, Rossler W, Obermayer M, Bickmeyer U (2006). Functional neuroanatomy of the rhinophore of *Aplysia punctata*. Front. Zool..

[45] Moroz LL, Romanova DY, Kohn AB (2021). Neural versus alternative integrative systems: Molecular insights into origins of neurotransmitters. Phil. Trans. R. Soc. B.

[46] Illes P (2021). Update of P2X receptor properties and their pharmacology: IUPHAR review 30. Br. J. Pharmacol..

[47] Knudsen B, Kohn AB, Nahir B, McFadden CS, Moroz LL (2006). Complete DNA sequence of the mitochondrial genome of the sea-slug, Aplysia californica: Conservation of the gene order in Euthyneura. Mol. Phylogenet. Evol..

[48] Zimmer-Faust RK (1993). ATP: A potent prey attractant evoking carnivory. Limnol. Oceanogr..

[49] Reklow RJ (2019). The purinome and the prebotzinger complex: A menage of unexplored mechanisms that may modulate/shape the hypoxic ventilatory response. Front. Cell. Neurosci..

[50] Burnstock G, Verkhratsky A (2009). Evolutionary origins of the purinergic signalling system. Acta Physiol. (Oxf).

[51] Jacobson KA (2020). Update of P2Y receptor pharmacology: IUPHAR Review 27. Br. J. Pharmacol..

[52] Pastor-Anglada M, Perez-Torras S (2018). Who is who in adenosine transport. Front. Pharmacol..

[53] Pastor-Anglada M, Perez-Torras S (2018). Emerging roles of nucleoside transporters. Front. Pharmacol..

[54] Pastor-Anglada M, Urtasun N, Perez-Torras S (2018). Intestinal nucleoside transporters: Function, expression, and regulation. Compr. Physiol..

[55] Hasuzawa N, Moriyama S, Moriyama Y, Nomura M (2020). Physiopathological roles of vesicular nucleotide transporter (VNUT), an essential component for vesicular ATP release. Biochim. Biophys. Acta Biomembr..

[56] Sawada K (2008). Identification of a vesicular nucleotide transporter. Proc. Natl. Acad. Sci. USA.

[57] Abrams, T. A. & Sossin, W. in *The Oxford Handbook of Invertebrate Neurobiology* (ed J.H. Byrne) 123–150 (2919).

[58] Zimmermann H, Zebisch M, Strater N (2012). Cellular function and molecular structure of ecto-nucleotidases. Purinergic Signal.

[59] Yegutkin GG (2014). Enzymes involved in metabolism of extracellular nucleotides and nucleosides: functional implications and measurement of activities. Crit. Rev. Biochem. Mol. Biol..

[60] Moroz LL (2006). Neuronal transcriptome of *Aplysia*: neuronal compartments and circuitry. Cell.

[61] Moroz, L. L. & Kohn, A. B. Do different neurons age differently? Direct genome-wide analysis of aging in single identified cholinergic neurons. *Front Aging Neurosci.* **2**, 1. doi:10.3389/neuro.24.006.2010 (2010). 10.3389/neuro.24.006.2010 PMC2910937 20725513

[62] Kohn AB, Moroz TP, Barnes JP, Netherton M, Moroz LL (2013). Single-cell semiconductor sequencing. Methods Mol. Biol..

[63] Puthanveettil SV (2013). A strategy to capture and characterize the synaptic transcriptome. Proc. Natl. Acad. Sci. U S A.

[64] Moroz LL, Kohn AB (2013). Single-neuron transcriptome and methylome sequencing for epigenomic analysis of aging. Methods Mol. Biol..

[65] Jezzini SH, Bodnarova M, Moroz LL (2005). Two-color in situ hybridization in the CNS of *Aplysia californica*. J. Neurosci. Methods.

[66] Moroz, L. L. & Kohn, A. B. in *In Situ Hybridization Methods, Neuromethods* Vol. 99 (ed Giselbert Hauptmann) 293–317 (Springer Science+Business Media, 2015).

[67] Trapnell C (2010). Transcript assembly and quantification by RNA-Seq reveals unannotated transcripts and isoform switching during cell differentiation. Nat. Biotechnol..

[68] Kim D (2013). TopHat2: accurate alignment of transcriptomes in the presence of insertions, deletions and gene fusions. Genome Biol..

[69] Berman H, Henrick K, Nakamura H (2003). Announcing the worldwide Protein Data Bank. Nat. Struct. Biol..

[70] *Schrodinger, L. The PyMOL Molecular Graphics System, Version 1.3r1*, https://www.scirp.org/(S(vtj3fa45qm1ean45vvffcz55))/reference/ReferencesPapers.aspx?ReferenceID=1571978 (2010).

[71] DeLano, W. L. *The PyMOL molecular graphics system*, http://www.pymol.org (2002).

[72] Kelley LA, Mezulis S, Yates CM, Wass MN, Sternberg MJ (2015). The Phyre2 web portal for protein modeling, prediction and analysis. Nat. Protoc..

[73] Moroz LL, Gyori J, Salanki J (1993). NMDA-like receptors in the CNS of molluscs. NeuroReport.

[74] Wagner GP, Kin K, Lynch VJ (2012). Measurement of mRNA abundance using RNA-seq data: RPKM measure is inconsistent among samples. Theory Biosci..

[75] Zhao S, Ye Z, Stanton R (2020). Misuse of RPKM or TPM normalization when comparing across samples and sequencing protocols. RNA.

[76] Abrams ZB, Johnson TS, Huang K, Payne PRO, Coombes K (2019). A protocol to evaluate RNA sequencing normalization methods. BMC Bioinformatics.

[77] North, R. A. P2X receptors. *Philos. Trans. R Soc. Lond. B Biol. Sci.* **371**, doi:10.1098/rstb.2015.0427 (2016). 10.1098/rstb.2015.0427 PMC4938027 27377721

