# Supplementary material for: ATP signaling in the integrative neural center of Aplysia californica
Source: Sci Rep. 2021 Mar 9;11:5478. doi: 10.1038/s41598-021-84981-5 (PMC7943599; doi:10.1038/s41598-021-84981-5)
Supplement: Supplementary file 1 — Supplementary Information [file 41598_2021_84981_MOESM1_ESM.pdf]

SUPPLEMENTARY INFORMATION

ATP signaling in the integrative neural center of *Aplysia californica*

János Györi[1¶], Andrea B. Kohn[2¶], Daria Y. Romanova[3], Leonid L. Moroz[2,4¶*]

[1]Centre for Ecological Research, Department of Experimental Zoology, Balaton Limnological Institute, H-8237 Tihany, Hungary; [2]Whitney Laboratory for Marine Bioscience, University of Florida, St. Augustine, FL, 32080,USA; [3]Institute of Higher Nervous Activity and Neurophysiology, Moscow, 117485, Russia; [4]Departments of Neuroscience and McKnight Brain Institute, University of Florida, Gainesville, FL, 32610,USA

¶-equal contribution

*****Corresponding author

Emails:moroz@whitney.ufl.edu
https://orcid.org/0000-0002-1333-3176

Running title: ATP modulation in *Aplysia*

Supplementary Figures

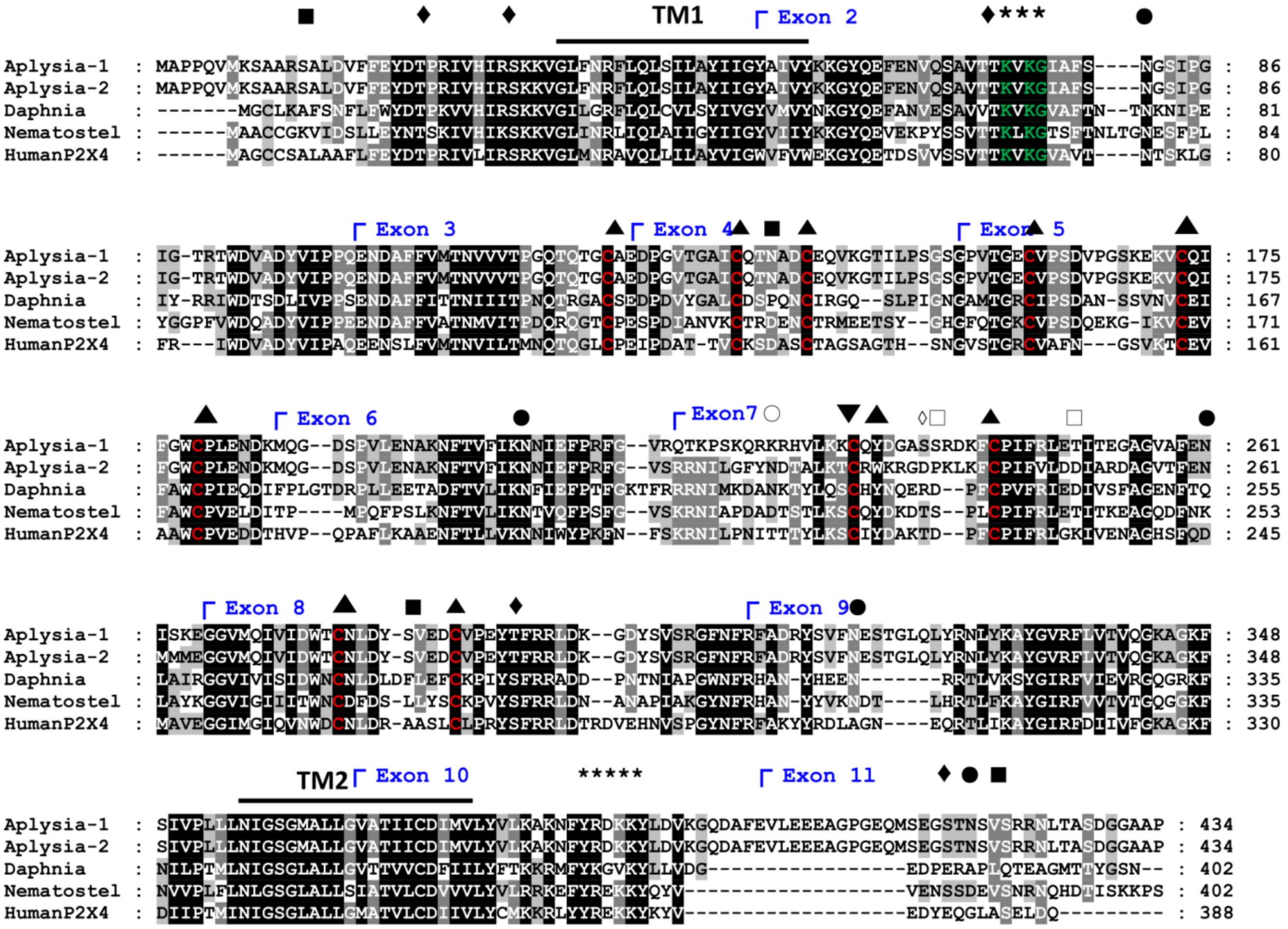

Figure 1S. Alignment of predicted amino acid sequences for the P2X receptor subfamily. The predicted amino acid sequences for *Ac*P2X and *Ac*P2X$_b$ were aligned with P2X$_4$ like receptors as a mammalian reference receptor of comparable size. The names and accession numbers for sequences used are found in Table 1S except for *Daphnia pulex*, EFX89098.1. The exon/intron boundaries, as well as protein motifs, are based on the genomic or predicted protein sequence of *Ac*P2X. The key for marked predicted motifs or amino acid residues is as follows: **-** Transmembrane region; ● N-glycosylation site; ♦ PKC phosphorylation site; ■ CK2 phosphorylation site; ◊□○ Present only in *Ac*P2X with the same designation as filled in symbols; ▼ Present only in *Ac*P2X$_b$ (PKC phosphorylation site); ▲ Conserved cysteines (red); *** Predicted ATP binding site [1] (green); ***** Predicted trafficking motif[2]; ┌ Exon/Intron Boundaries (blue). Exon 7 is the only difference between the two *Aplysia* P2X isoforms. Both *Ac*P2X and *Ac*P2X$_b$ contain five predicted N-glycosylation sites fond mainly in the putative extracellular loop region, *Ac*P2X contains an additional site in the extracellular loop. Secondary motifs were determined with Prosite[3]. Both predicted isoforms contain seven putative Protein kinase C (PKC) phosphorylation sites; each has one site with a different location due to the splice region. Of the predicted PKC sites, both *Aplysia* subunits contain the critical site potentially involved in desensitization along with the conserved positively charged amino acid residues also involved in desensitization[4,5]. *Ac*P2X and *Ac*P2X$_b$ contain four Casein kinase II phosphorylation (CK2) sites, with *Ac*P2X having an additional two sites in its extracellular loop.

Human *P2X$_4$* and mouse *P2X$_4$* have 12 coding exons[6]. Both isoforms of *Aplysia P2X* receptor genes have 11 coding exons in which the 9 and 10 exons are fused compared to vertebrates; however, they still generate a conserved exon/intron boundary. The *Aplysia* genome assembly did not produce a single contig where the *P2X* receptors were detected. The two other invertebrate *P2X*-like receptor sequences from *Daphnia* and *Nematostella* only have 8 coding exons, with 4 exons likely being fused. However, the exon/intron boundaries of those present in their genomes are still conserved.

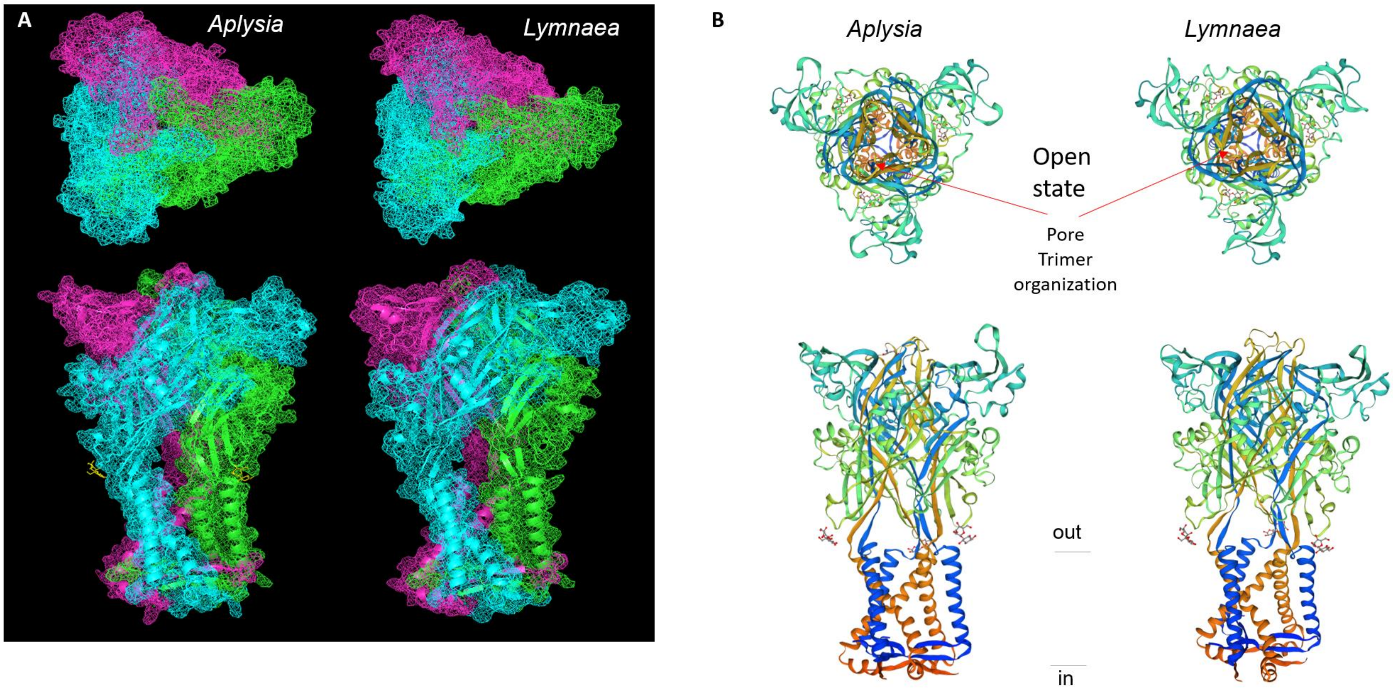

Fig. 2S. The hypothetical 3D-structure of P2X receptors for *Aplysia californica* and *Lymnaea stagnalis*. A and B. 3D modeling for P2X receptors of *A. californica* and *L. stagnalis* with a predicted open state (model PDB: 5svk), see details in[7]. Alternative models of P2X receptors were generated using PyMol (The PyMol Molecular Graphics System, Version 1.8.6.0 Schrödinger, LLC) and Phyre2 software[7-10].

Supplementary Tables

Table 1S Sequences used in phylogenetic analysis.

Species	**Accession number**	**Name on Tree**
Homo sapiens	NP_002549.1	*Homo_P2RX1*
Homo sapiens	NP_733782.1	*Homo_P2RX2*
Homo sapiens	NP_002550.2	*Homo_P2RX3*
Homo sapiens	NP_002551.2	*Homo_P2RX4*
Homo sapiens	NP_002552.2	*Homo_P2RX5*
Homo sapiens	NP_005437.2	*Homo_P2RX6*
Homo sapiens	NP_002553.3	*Homo_P2RX7*
Danio rerio	NP_945333.1	*Danio_P2RX1*
Danio rerio	NP_945334.1	*Danio_P2RX2*
Danio rerio	NP_945337.3	*Danio_P2RX3b*
Danio rerio	NP_705939.1	*Danio_P2RX4a*
Danio rerio	NP_919394.1	*Danio_P2RX5*
Danio rerio	NP_945335.1	*Danio_P2RX7*
Danio rerio	NP_945336.1	*Danio_P2RX8*
Saccoglossus kowalevskii	NP_001161641.1	*Aaccoglossus_P2RXA*
Saccoglossus kowalevskii	XP_006821899.1	*Saccoglossus_P2RXB*
Strongylocentrotus purpuratus	XP_001181725.1	*Strongylocentrotus_P2RXA*
Strongylocentrotus purpuratus	XP_001181346.1	*Strongylocentrotus_P2RXB*
Schistosoma mansoni	CAH04147.1|	*Schistosoma_P2XR*
Capitella teleta	ELU13670.1	*Capitella_P2RX*
Octopus vulgaris	QHX41545.1	*Octopus_P2RX*
Crassostrea virginica	XP_022345450.1	*Crassostrea_P2RX*
Lymnaea stagnalis	AFV69113.1	*Lymnaea_P2RX*
Elysia chlorotica	RUS81419.1	*Elysia_P2RX*
Lottia gigantea	XP_009048327.1	*Lottia_P2RX*
Aplysia californica	NP_001191558.1	*Aplysia_P2RX*
Nematostella vectensis	XP_032239188.1	*Nematostella_P2RXA*
Nematostella vectensis	XP_032238507.1	*Nematostella_P2RXB*
Amphimedon queenslandica	XP_003385281.1	*Amphimedon_P2RXA*
Amphimedon queenslandica	XP_003384136.1	*Amphimedon_P2RXB*
Trichoplax sp. H2	RDD47347.1	*TrichoplaxH2_P2RXA*
Trichoplax sp. H2	RDD40737.1	*TrichoplaxH2_P2RXB*
Mnemiopsis leidyi	ML088815a	*Mnemiopsis_P2RX*
Pleurobrachia bachei	ADV31315	*Pleurobrachia_P2RX*
Capsaspora owczarzaki	KJE88670.1	*Capsaspora_P2RX*
Monosiga brevicollis MX1	EDQ92249.1	*Monosiga_P2RX*
Dictyostelium discoideum AX4	XP_645378.1	*Dictyostelium_P2RX*

Table 2S. RNA-seq samples for *Aplysia californica*

**SRA study SRP001185**	**SRA Run**
Early Cleavage	**SRX268443**
Gastrulation	**SRX268442**
3 Day	**SRX268440**
4 Day early trochophora	**SRX268437**
9 Day veliger	**SRX268438**
12 Day hatching veliger	**SRX268439**
**BioProject PRJNA77701**	
Chemoreceptive tissue	**SRX109676**
Heart	**SRX109675**
Hepatopancreas	**SRX109682**
Gills	**SRX109674**
Salivary Glands	**SRX109678**
CNS	**SRX109680**
Muscle	**SRX109681**
Other digestive parts	**SRX109679**
Ovotestis	**SRX109677**
Hermaphroditic glands and reproductive	**SRX109683**

References for Supplementary Information

1 Chataigneau, T., Lemoine, D. & Grutter, T. Exploring the ATP-binding site of P2X receptors. *Frontiers in cellular neuroscience* **7**, 273, doi:10.3389/fncel.2013.00273 (2013).

2 Chaumont, S., Jiang, L. H., Penna, A., North, R. A. & Rassendren, F. Identification of a trafficking motif involved in the stabilization and polarization of P2X receptors. *J Biol Chem* **279**, 29628-29638, doi:10.1074/jbc.M403940200 (2004).

3 Sigrist, C. J. *et al.* New and continuing developments at PROSITE. *Nucleic Acids Res* **41**, D344-347, doi:10.1093/nar/gks1067 (2013).

4 Werner, P., Seward, E. P., Buell, G. N. & North, R. A. Domains of P2X receptors involved in desensitization. *Proceedings of the National Academy of Sciences of the United States of America* **93**, 15485-15490 (1996).

5 Ennion, S., Hagan, S. & Evans, R. J. The role of positively charged amino acids in ATP recognition by human P2X1 receptors. *J Biol Chem* **275**, 35656 (2000).

6 Illes, P. *et al.* Update of P2X receptor properties and their pharmacology: IUPHAR Review 30. *Br J Pharmacol* **178**, 489-514, doi:10.1111/bph.15299 (2021).

7 Kelley, L. A., Mezulis, S., Yates, C. M., Wass, M. N. & Sternberg, M. J. The Phyre2 web portal for protein modeling, prediction and analysis. *Nat Protoc* **10**, 845-858, doi:10.1038/nprot.2015.053 (2015).

8 *Schrodinger, L. The PyMOL Molecular Graphics System, Version 1.3r1*, <https://www.scirp.org/(S(vtj3fa45qm1ean45vvffcz55))/reference/ReferencesPapers.aspx?ReferenceID=1571978> (2010).

9 Berman, H., Henrick, K. & Nakamura, H. Announcing the worldwide Protein Data Bank. *Nat Struct Biol* **10**, 980, doi:10.1038/nsb1203-980 (2003).

10 DeLano, W. L. *The PyMOL molecular graphics system*, <http://www. pymol. org. > (2002).
